# Comparative study: Vonoprazan and proton pump inhibitors in *Helicobacter pylori* eradication therapy

**DOI:** 10.3748/wjg.v23.i4.668

**Published:** 2017-01-28

**Authors:** Kouichi Sakurai, Hiroko Suda, Yumi Ido, Takayuki Takeichi, Ayako Okuda, Kiwamu Hasuda, Masahiro Hattori

**Affiliations:** Kouichi Sakurai, Hiroko Suda, Yumi Ido, Takayuki Takeichi, Ayako Okuda, Kiwamu Hasuda, Masahiro Hattori, Hattori Clinic, Chuo-ku, Kumamoto 860-0004, Japan

**Keywords:** *Helicobacter pylori*, Eradication treatment, Vonoprazan, Proton pump inhibitors, Adverse event, Smoking

## Abstract

**AIM:**

To compare the effectiveness and safety of vonoprazan-based therapy with proton pump inhibitor (PPI)-based therapies to treat *Helicobacter pylori* (*H. pylori*).

**METHODS:**

We retrospectively analysed data from first-line (vonoprazan or PPI with 200 mg clarithromycin and 750 mg amoxicillin twice daily for 7 d) (*n* = 1353) and second-line (vonoprazan or PPI with 250 mg metronidazole and 750 mg amoxicillin twice daily for 7 d) (*n* = 261) eradication treatments for *H. pylori* -positive patients with associated gastrointestinal diseases from April 2014 to December 2015 at Hattori Clinic, Japan. The primary endpoint was the eradication rate, which was assessed with a full analysis set. The secondary endpoints were adverse events and related factors.

**RESULTS:**

After the first-line treatments, the eradication rates for vonoprazan, esomeprazol, rabeprazole, and lansoprazole were 87.9% (95%CI: 84.9%-90.5%), 71.6% (95%CI: 67.5%-75.5%), 62.9% (95%CI: 52.0%-72.9%), and 57.3% (95%CI: 50.4%-64.1%), respectively. The vonoprazan eradication rate was significantly higher than that of the PPIs (*P* < 0.01). Interestingly, smoking did not affect the *H. pylori* eradication rate in the vonoprazan group (*P* = 0.34), whereas it decreased the rates in the PPI groups (*P* = 0.013). The incidence of adverse events in the vonoprazan group was not different from the PPI group (*P* = 0.054), although the vonoprazan group exhibited a wider range of adverse events. Vonoprazan-based triple therapy was highly effective as a second-line treatment, with an eradication rate similar to that of PPI-based therapy.

**CONCLUSION:**

Vonoprazan might be superior to PPIs in first-line *H. pylori* therapy, particularly for smokers. However, caution is required due to possible adverse events.

**Core tip:** Because the *Helicobacter pylori* (*H. pylori*) eradication rate of conventional proton pump inhibitor (PPI)-based treatment has decreased because clarithromycin-resistant strains have appeared in recent years, a new treatment strategy is required. Vonoprazan is a novel potassium -competitive acid blocker that has strong, long-lasting effects, but few studies have investigated its efficacy against *H. pylori*. We compared vonoprazan-based therapy and PPI-based therapy as first-line and second-line treatments. Vonoprazan-based therapy was superior to PPI-based therapy, particularly for smokers, but adverse events (AEs) due to vonoprazan occurred more frequently. Vonoprazan-based therapy is a potentially efficacious treatment, but it should be used with caution due to possible AEs.

## INTRODUCTION

*Helicobacter pylori* (*H. pylori*) contributes to upper gastrointestinal diseases, such as chronic gastritis, peptic ulcers, mucosa-associated lymphoid tissue (MALT) lymphoma, and gastric cancer[1-3]. Proton pump inhibitor (PPI)-based *H. pylori* eradication therapy has been shown to be effective for treatment of *H. pylori*-related diseases[4].

In Japan, the first-line regimen consists of triple therapy with a PPI (omeprazole, rabeprazole, lansoprazole, or esomeprazole), amoxicillin (AMPC), and clarithromycin (CAM) for 7 d. As of 2000, the cost of this therapy for patients with gastric or duodenal ulcers has been covered under Japan’s national health insurance[5]. After the approval of the first regimen, the second-line treatment, in which CAM is replaced by metronidazole (MNZ), was also approved in 2007. Dating from February 2013, *H. pylori* eradication therapy was expanded to include patients with *H. pylori* infection-associated gastritis to prevent gastric cancer.

However, the eradication rate with the first-line treatment has reportedly decreased due to the increase of CAM-resistant strains in recent years[5-7]. Therefore, a more effective strategy is required for CAM-resistant patients.

Vonoprazan is a novel potassium-competitive acid blocker (P-CAB) and to a new class of gastric acid-suppressive agents[8]. P-CABs, which block H^+^, K^+^ ATPase in a competitive and reversible manner, result in stronger and more sustained acid suppression than PPIs[9]. Alteration of the intragastric pH, to a higher pH with a lower percentage of time spent < pH 4, is crucial in *H. pylori* eradication therapy[10]. Therefore, P-CAB-based triple therapy should be more efficient than PPI-based therapy for *H. pylori*-infected patients, including CAM-resistant patients. A phase III randomized, double-blind study showed that vonoprazan (P-CAB)-based treatment was effective in both first- and second-line *H. pylori* eradication therapy compared to treatment with lansoprazol[11].

In this study, we evaluated the clinical effectiveness and safety of vonoprazan-based *H. pylori* eradication therapy and compared it to that of conventional PPI-based therapy in clinical practice.

## MATERIALS AND METHODS

### Patients and study design

This study was conducted in a single institution (Hattori Clinic). We retrospectively examined data from patients administered first- and/or second-line eradication therapy.

*H. pylori*-positive patients diagnosed via a high-resolution endoscope (GIF260, 290 series; Olympus, Tokyo, Japan) with gastric ulcer and/or ulcer scar (GU/GUs), duodenal ulcer and/or ulcer scar (DU/DUs), gastroduodenal ulcer and/or ulcer scar (GDU/GDUs), gastric MALT lymphoma, post-endoscopic submucosal dissection (post ESD) for early gastric cancer or atrophic gastritis from April 2014 to December 2015 at Hattori Clinic were enrolled in this study.

The exclusion criteria were as follows: less than 20 years of age; past history of total gastrectomy; history of drug allergy to PPIs, AMPC, CAM, or MNZ; clinically significant disease (hepatic, renal or cardiac disease); and pregnancy.

The presence of *H. pylori* at admission and after first- and second-line eradication therapy was confirmed with the ^13^C-urea breath test (UBT). The cut-off value was 2.5‰. Confirmation of eradication by UBT was performed no less than 8 wk after eradication treatment was completed. UBT-negative patients whose endoscopic findings showed gastric atrophy received an additional stool antigen test.

*H. pylori*-positive patients received one of the following first-line treatments: (1) vonoprazan (VPZ) group; 20 mg vonoprazan, 200 mg CAM, and 750 mg AMPC twice daily for 7 d; (2) esomeprazole (EPZ) group; 20 mg esomeprazole, 200 mg CAM, and 750 mg AMPC twice daily for 7 d; (3) rabeprazole (RPZ) group; 10 mg rabeprazole, 200 mg CAM, and 750 mg AMPC twice daily for 7 d; (4) lansoprazole (LPZ) group; 30 mg lansoprazole, 200 mg CAM, and 750 mg AMPC twice daily for 7 d. For the second-line treatment, 200 mg CAM was replaced with 250 mg MNZ and a selected acid blocker medication depended on the attending doctor’s discretion.

Adverse events (AEs) were defined as undesirable medical symptoms or conditions after the beginning of the treatment, which were interrogated directly by each investigator.

The primary endpoint was eradication rate. The secondary endpoints were AEs and related factors.

Subgroup analyses of demographic and clinical characteristics, including presence of endoscopic gastric atrophy, age, gender, alcohol consumption, body mass index (BMI), and smoking, were also conducted. The Kimura-Takemoto classification was used to classify atrophic gastritis[12].

This study was conducted in accordance with the Declaration of Helsinki, and the research protocol was approved by the institutional review board of the Hattori Clinic.

### Statistical analysis

The eradication rate was evaluated with a full analysis set (FAS) and calculated with 95%CI. In the FAS analysis, patients who were lost during follow-up and who did not comply with the protocol were excluded.

Statistical analysis of eradication rate among the four regimens was assessed using the χ^2^ test. Patient characteristics among the four groups were assessed *via* Fisher’s exact test and the χ^2^ test. Factors associated with treatment failure were assessed by logistic regression analysis. *P* values < 0.05 were considered to be statistically significant.

## RESULTS

### Patient characteristics

In total, 1353 patients completed the first-line treatment protocol. The baseline characteristics and demographics of patients in this study were presented in Table 1. Most patients (*n* = 1169) were diagnosed with *H. pylori*-infected gastritis without any other lesions or diseases. Others diagnoses were GU/GUs (*n* = 65), DU/DUs (*n* = 105), GDU/GDUs (*n* = 8), MALT lymphoma (*n* = 2), and post ESD for early gastric cancer (*n* = 4). The patients were treated with VPZ (*n* = 546), EPZ (*n* = 507), RPZ (*n* = 89), or LPZ (*n* = 211). Demographic and other baseline characteristics for all the patients receiving the four regimens were not significantly different with regard to age, sex, and upper gastrointestinal diseases. In total, 261 patients completed the second-line treatment protocol. Demographic and other baseline characteristics in the second-line treatment were also shown in Table 1 and there were not significant differences in all of them.

**Table 1:** Baseline and demographic characteristics of patients in this study

	**First-line eradication therapy**					**Second-line eradication therapy**				
	**VPZ group**	**PPI group**	**EPZ group**	**RPZ group**	**LPZ group**	**VPZ group**	**PPI group**	**EPZ group**	**RPZ group**	**LPZ group**
	***n* = 546**	***n* = 807**	***n* = 507**	***n* = 89**	***n* = 211**	***n* = 76**	***n* = 185**	***n* = 104**	***n* = 24**	***n* = 57**
Age, mean ± SD, yr	57.4 ± 11.8	56.7 ± 12.8	56.9 ± 11.6	60.7 ± 11.2	56.1 ± 12.1	56.9 ± 12.8	56.0 ± 12.6	57.5 ± 12.5	58.2 ± 12.2	58.1 ± 12.3
Sex, *n* (%)										
Male	225 (41.2)	318 (39.4)	193 (38.1)	35 (39.3)	90 (42.7)	30 (39.5)	71 (38.4)	39 (37.5)	9 (39.3)	23 (40.4)
Female	321 (58.8)	489 (60.6)	314 (61.9)	54 (60.7)	121 (57.3)	46 (60.5)	114 (61.6)	65 (62.5)	15 (60.7)	34 (59.6)
Indication										
GU(s)	32	33	18	4	11	3	7	6	0	1
DU(s)	37	68	36	8	24	5	13	5	2	6
GDU(s)	4	4	2	1	1	1	0	0	0	0
MALT lymphoma	0	2	0	0	2	0	1	0	0	1
Post ESD	1	3	2	0	1	1	0	0	0	0
Atrophic gastritis	472	697	449	76	172	66	164	93	22	49

GU/GUs: Gastric ulcer and/or ulcer scar; DU/DUs: Duodenal ulcer and/or ulcer scar; GDU/GDUs: Gastroduodenal ulcer and/or ulcer scar; MALT: Mucosa-associated lymphoid tissue; ESD: Endoscopic submucosal dissection; VPZ: Vonoprazan; PPI: Proton pump inhibitor; EPZ: Esomeprazole; RPZ: Rabeprazole; LPZ: Lansoprazole.

### Eradication rates

**Figure 1:**
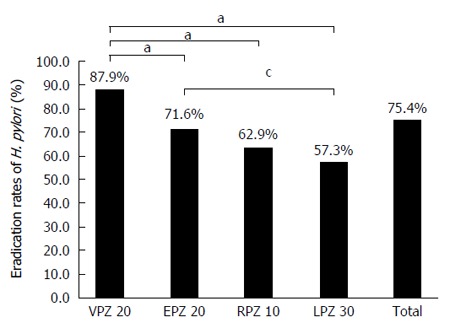
*Helicobacter pylori* eradication rates (full analysis set) for vonoprazan, esomeprazole, rabeprazole, and lansoprazole in first-line triple therapy. The eradication rate was significantly higher in the VPZ group than that in the EPZ, RPZ, and LPZ groups (^a^*P* < 0.05); ^c^*P* < 0.05 *vs* lansoprazole. VPZ 20: 20 mg VPZ, 200 mg CAM, and 750 mg AMPC twice a day for 1 wk. EPZ 20: 20 mg EPZ, 200 mg CAM, and 750 mg AMPC twice a day for 1 wk. RPZ 10: 10 mg RPZ, 200 mg CAM, and 750 mg AMPC twice a day for 1 wk. LPZ 30: 30 mg LPZ, 200 mg CAM, and 750 mg AMPC twice a day for 1 wk. VPZ: Vonoprazan; EPZ: Esomeprazole; CAM: Clarithromycin; AMPC: Amoxicillin; RPZ: Rabeprazole; LPZ: Lansoprazole; H. pylori: Helicobacter pylori.

FAS analysis indicated that the first-line treatment eradication rate was 87.9% (95%CI: 84.9%-90.5%) in the VPZ group, 71.6% (95%CI: 67.5%-75.5%) in the EPZ group, 62.9% (95%CI: 52.0%-72.9%) in the RPZ group, and 57.3% (95%CI: 50.4%-64.1%) in the LPZ group (Figure 1). The eradication rate achieved in the VPZ group was significantly higher than that in the other three groups ( Table 2).

**Table 2:** Univariate analysis of predictors for successful Helicobacter pylori first-line eradication

	**VPZ group**				**PPI group**			
	**Eradication rate**	**OR**	**95%CI**	***P* value**	**Eradication rate**	**OR**	**95%CI**	***P* value**
Sex								
Male	90.2%	1			67.0%	1		
Female	86.3%	0.682	0.396-1.174	0.17	66.9%	0.995	0.737-1.343	0.97
Age (yr)								
< 60	86.6%	1			74.1%	1		
≥ 60	87.9%	1	0.598-1.673	1	76.7%	1.155	0.860-1.551	0.34
BMI (kg/m^2^)								
< 25	88.4%	1			69.1%	1		
≥ 25	86.5%	0.843	0.464-1.530	0.57	68.8%	0.987	0.683-1.435	0.94
Smoking								
No	87.3%	1			68.1%	1		
Yes	91.3%	1.412	0.583-3.420	0.44	56.6%	0.611	0.412-0.906	0.01
Alcohol								
Non-drinker	88.1%	1			67.7%	1		
Drinker	87.4%	0.937	0.555-1.580	0.81	67.3%	0.984	0.716-1.352	0.92
Atrophy								
Closed type	87.9%	1			65.6%	1		
Open type	89.1%	1.130	0.619-2.069	0.69	65.6%	0.998	0.708-1.408	0.99

VPZ: Vonoprazan; PPI: Proton pump inhibitor.

**Figure 2:**
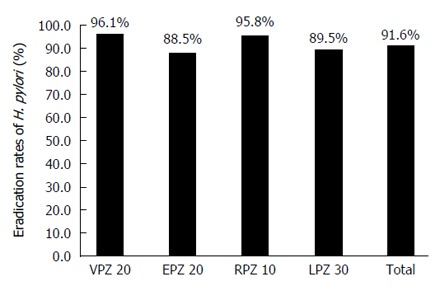
*Helicobacter pylori* eradication rates (full analysis set) for vonoprazan, esomeprazole, rabeprazole, and lansoprazole in second-line triple therapy. The eradication rates of the four second-line therapies were not significantly different. VPZ 20: 20 mg VPZ, 250 mg MNZ, and 750 mg AMPC twice a day for 1 wk; EPZ 20: 20 mg EPZ, 250 mg MNZ, and 750 mg AMPC twice a day for 1 wk; RPZ 10: 10 mg RPZ, 250 mg MNZ, and 750 mg AMPC twice a day for 1 wk; LPZ 30: 30 mg LPZ, 250 mg MNZ, and 750 mg AMPC twice a day for 1 wk. VPZ: Vonoprazan; MNZ: Metronidazole; AMPC: Amoxicillin; EPZ: Esomeprazole; RPZ: Rabeprazole; LPZ: Lansoprazole; H. pylori: Helicobacter pylori.

The eradication rates for the second-line treatment were 96.1% (95%CI: 88.9%-99.2%) in the VPZ group, 88.5% (95%CI: 80.7%-93.9%) in the EPZ group, 95.8% (95%CI: 78.9%-99.9%) in the RPZ group, and 89.5% (95%CI: 78.5%-96.0%) in the LPZ group (Figure 2), and there were no significant differences among the four groups.

We evaluated age, extent of atrophic gastritis, sex, BMI, alcohol abuse, and smoking habits as predictive factors for successful *H. pylori* eradication in the first- and second-line treatments among the four groups. For the first-line treatment, smoking alone showed a significant difference between the four groups by univariate analyses. Thus, smoking habits decreased *H. pylori* eradication rates in the three PPI groups, whereas eradication was unaffected in the VPZ group (Table 2). There were no predictive factors observed for second-line treatment and there were no predictive factors demonstrated in both the first-line treatment and the second-line treatment by multivariate analyses (data not shown).

### AEs

The AE incidence during first-line eradication therapy was 11.2% in the VPZ group compared with 7.7% in the EPZ group, 10.1% in the RPZ group, and 5.7% in the LPZ group. The most frequently observed AE was diarrhoea/soft stool. There was no significant difference in AE incidence between the VPZ group and the PPI groups (*P* = 0.054). However, the VPZ group showed a wider range of AEs including appetite loss, headache, fever and haematuria. The AE incidence for second-line eradication therapy was also not significantly different, and diarrhoea/soft stool occurred most frequently, similar to first-line eradication therapy (Table 3). None of the patients showed serious and critical AEs in during either the first or second-line therapy and none of the patients discontinued *H. pylori* eradication treatment because of AEs.

**Table 3:** Adverse events in first- and second-line eradication therapies *n* (%)

	**First-line eradication therapy**				**Second-line eradication therapy**			
	**VPZ**	**EPZ**	**RPZ**	**LPZ**	**VPZ**	**EPZ**	**RPZ**	**LPZ**
Adverse events	61 (11.2)	39 (7.7)	9 (10.1)	12 (5.7)	6 (7.9)	14 (13.5)	3 (12.5)	7 (12.3)
Diarrhoea/soft stool	29 (47.5)	25 (64.1)	5 (55.6)	8 (66.7)	4 (66.7)	6 (43.0)	3 (100)	5 (71.4)
Eruption	7 (11.5)	3 (7.7)	2 (22.2)	2 (16.7)	0	3 (21.4)	0	0
Constipation	6 (10.0)	3 (7.7)	0	0				
Dysgeusia	3 (5.0)	3 (7.7)	1 (11.1)	0	0	1 (7.1)	0	1 (14.3)
Nausea and vomiting	2 (3.3)	2 (5.1)	0	1 (8.3)	0	1 (7.1)	0	0
Abdominal pain	5 (8.2)	2 (5.1)	0	0	1 (16.7)	2 (14.3)	0	0
Appetite loss	3 (5.0)	0	0	0				
General fatigue	2 (3.3)	1 (2.6)	0	0	1 (16.7)	0	0	0
Heartburn	1 (1.6)	0	0	1 (8.3)				
Headache	1 (1.6)	0	0	0	0	0	0	1 (14.3)
Fever	1 (1.6)	0	0	0				
Flatulence	0	0	1 (11.1)	0				
Haematuria	1 (1.6)	0	0	0				
Vertigo					0	1 (7.1)	0	0

VPZ: Vonoprazan group; EPZ: Esomeprazole group; RPZ: Rabeprazole group; LPZ: Lansoprazole group.

## DISCUSSION

We compared the efficacies and safety profiles of vonoprazan and three PPIs (esomeprazole, rabeprazole, and lansoprazole) used for *H. pylori* eradication and found vonoprazan to be superior to PPIs as a first-line therapy (*P* < 0.01).

Several factors, such as cytochrome P450 2C19 (CYP2C19) polymorphisms, antibiotic susceptibility, smoking habits, and patient compliance, are known to cause *H. pylori* eradication failure[13]. Insufficient gastric acid inhibition and *H. pylori* antibiotic resistance are major factors underlying *H. pylori* eradication failure[13].

*H. pylori* eradication is dependent on the maintenance of a near neutral gastric pH throughout the day[11]. Maintenance of a gastric pH > 5 is necessary for *H. pylori* to replicate. Additionally, acid suppression has been reported to enhance antibiotic stability and bacterial sensitivity[14]. Therefore, adequate acid suppression is essential for *H. pylori* eradication therapy.

Vonoprazan is a highly effective anti-acid drug compared to PPIs. Sakurai et al[15] showed that vonoprazan suppressed acid secretion more rapidly and persistently than two PPIs, esomeprazole and rabeprazole. The authors showed that even on the first day of administration, the mean pH for vonoprazan was above 5, in contrast to the pH induced by PPIs, and the 24 h pH 4 holding-time (acid-inhibitory effect) of vonoprazan greatly exceeded that of the PPIs[15]. Considering the significance of acid suppression for *H. pylori* eradication therapy, vonoprazan could be effective for resolving *H. pylori* eradication failure.

The acid-inhibitory effect of PPIs is affected by CYP2C19 polymorphisms, and for extensive CYP2C19 metabolizers, the acid-inhibitory effect of PPIs is decreased. The population frequency of poor, intermediate, extensive metabolizers in Japan is 18.8%, 43.8% and 35.5%, respectively[16].

Esomeprazole is less susceptible to CYP2C19 polymorphisms[17,18]. Indeed, the eradication rate of esomeprazole was significantly higher than that of lansoprazole in our study. We suggest that the eradication rates observed in our results correspond to the population frequency of genetic polymorphisms affecting the metabolism of the PPI. Vonoprazan is also unaffected by CYP2C19 polymorphisms because it is poorly metabolized by CYP2C19[19-21].

We hypothesized that the significantly higher eradication rate for vonoprazan compared to the other PPIs was caused by tolerance of CYP2C19 polymorphisms.

Drug interactions with antibiotics may also affect eradication rate. Comparing the pharmacokinetics of co-administration of vonoprazan, CAM plus AMPC and single administration of the drugs, co-administered vonoprazan and CAM resulted in a higher mean Cmax and area under the curve (AUC) compared with those of single administration. However, AMPC had no effect on Cmax and AUC[19-21]. Both CAM and vonoprazan are predominantly metabolized by CYP3A4 and have also been reported to be possible CYP3A4 inhibitors when used in combination. Thus, vonoprazan and CAM might mutually inhibit their metabolism, increasing the plasma concentrations of both CAM and vonoprazan. A phase III, randomized, double-blind, multicentre study on vonoprazan showed higher eradication rates with vonoprazan (82.0%) against CAM resistance compared to that of lansoprazole (40.0%)[10]; thus, we hypothesize that these pharmacological characteristics of vonoprazan will be advantageous for *H. pylori*-eradication therapy.

Although vonoprazan might be effective in *H. pylori* eradication therapy, AEs are still possible with this new drug. However, in our study, there was no significant difference in the incidence of AEs between vonoprazan and PPIs (*P* = 0.054). Notably, vonoprazan caused a wider variety of AEs compared to PPIs, although every AE was relatively mild and completely resolved after discontinuation of *H. pylori* eradication therapy. As we mentioned above, the plasma concentrations of CAM and vonoprazan might be increased during first-line treatment, making it likely that unexpected and more frequent AEs happen. Thus, vonoprazan-associated AEs need to be carefully monitored in the future.

A phase III study also showed an extremely high eradication rate (98%) for second-line triple therapy with vonoprazan, AMPC, and MNZ[8]. Correspondingly, our second-line eradication rate was 96.1% overall, and there were no significant differences in the second-line eradication rates and AEs between the three PPIs and vonoprazan. In the second-line treatment, CAM was replaced by MNZ, which is believed to be unaffected by CYP3A and CYP2C19 polymorphisms. Due to the original high eradication rates of second-line therapy with PPIs, addition of MNZ did not result in differences in the second-line eradication rates.

Tobacco smoking was also demonstrated to be a factor in *H. pylori* eradication failure; this is because smoking stimulates acid secretion[22], affects the metabolism of cytochrome P450[23], and decreases gastric blood flow and mucous secretion[24]. Hence, smoking reduces the delivery of antibiotics to the gastric mucosa. Most significantly, smoking stimulates acid secretion, which results in decreased intragastric pH. Indeed, Suzuki et al[25] reported that smoking increased the risk of *H. pylori* eradication failure. Interestingly, *H. pylori* eradication rates among patients in the VPZ group who smoked were unaffected in our study, whereas the eradication rates in the PPI groups decreased significantly. Thus, *H. pylori* therapy with vonoprazan was effective even for smoking patients; this was most likely because of strong acid suppression. Although smoking alone showed a significant difference between the four groups by univariate analyses, it was not a significant factor by multivariate analyses. We believe that this result might have arisen because we did not investigate drug sensitivity, and *H. pylori* resistance to CAM might be a major reason for treatment failure. It is necessary to investigate factors contributing to the success of *H. pylori* eradication treatment, including drug sensitivity, in the future.

This study was limited because it was retrospective and only performed at a single centre; moreover, we did not investigate drug sensitivity and CYP2C19 polymorphism. Triple therapy combining a PPI with AMPC and CAM generates an unacceptably low eradication rate in most of the world. Sequential therapy, quadruple therapy, concomitant therapy and high dose dual therapy are recommended in the era of increased CAM resistance[13]. However, although the resistance of *H. pylori* to CAM is increasing in Japan, only triple therapy with PPI, AMPC and CAM has been recognized under Japan’s national health insurance. Vonoprazan-based triple therapy has been available in Japan dating from February 2015. In our results, the vonoprazan eradication rate was high (87.9%), but it is not a satisfactory result regarding the efficacy of *H. pylori* eradication throughout the world. If vonoprazan -based triple therapy was provided to CAM -sensitive patients, the eradication rate might increase to over 90%. Therefore, it is necessary to investigate drug sensitivity before treatment in Japan. If the patients have CAM-resistant strains of *H. pylori*, they will require regimens of vonoprazan and different types and doses of antibiotics as well as different periods instead of CAM.

*H. pylori* eradication therapy is an effective treatment to help prevent gastric cancer. However, indiscriminate use of *H. pylori* eradication therapy might result in the expansion of antibiotic resistance instead of promoting gastric cancer prevention. Therefore, we need to select appropriate *H. pylori* eradication regimens by taking into consideration individual variations, such as antibiotic resistance and CYP2C19 polymorphism; however, the cost of these tests is not covered under Japan’s national health insurance scheme.

In conclusion, triple therapy with vonoprazan, AMPC, and CAM is superior to PPI-based therapy for first-line eradication, however possible AEs should be monitored.

## COMMENTS

### Background

*Helicobacter pylori* (*H. pylori*) eradication therapy is an effective treatment to help prevent gastric cancer. Triple therapy combining a proton pump inhibitor (PPI) with amoxicillin (AMPC), and clarithromycin (CAM) for *H. pylori* eradication is standard first-line therapy in Japan. However, the *H. pylori* eradication rate has decreased due to the increased prevalence of CAM resistance, thus a more effective strategy is required. Vonoprazan is a novel potassium -competitive acid blocker that has strong, long-lasting effects, but few studies have investigated its efficacy against *H. pylori* thus far. In this study, we retrospectively examined the effectiveness and safety of vonoprazan-based therapy compared with PPI-based therapies to treat *H. pylori*.

### Research frontiers

Triple therapy combining a PPI with AMPC and CAM provides unacceptably low eradication rates in most regions of the world. A good *H. pylori* therapy regimen with an eradication rate above 90% is needed. A more effective regimen is necessary in Japan.

### Innovations and breakthroughs

In this study, vonoprazan resulted in a significantly higher eradication rate (87.9%) than that of the three PPIs in the first-line treatment. There were no significant differences in the second-line eradication rates. Interestingly, *H. pylori* eradication rate for vonoprazan in smoking patients was similar to that of non-smoking patients, whereas the eradication rates with PPIs for smokers were decreased.

### Applications

It is necessary to investigate drug sensitivity before treatment because *H. pylori* CAM resistance is increasing in Japan. If the patients have CAM-resistant strains of *H. pylori*, treatment will require regimens of vonoprazan and different types and doses of antibiotics. Vonoprazan-containing triple therapy, quadruple therapy, sequential therapy and dual therapy that considers drug sensitivity will produce an excellent eradication rate.

### Peer-review

This manuscript is the retrospective study of *Helicobacter pylori* eradication using vonoprazan and conventional PPIs.
